# 2D/3D Perovskite Surface Passivation-Enabled High-Detectivity Near-Infrared Photodiodes

**DOI:** 10.3390/s25092740

**Published:** 2025-04-26

**Authors:** Xuefeng Huangfu, Junyu Chen, Gaohui Ge, Jianyu Li, Jiazhen Zhang, Qinhao Lin, Hao Xu, Shu Min Wang

**Affiliations:** 1 School of Physics, University of Electronic Science and Technology of China, Chengdu 611731, China; 2 Yangtze Delta Region Institute (Huzhou), University of Electronic Science and Technology of China, Huzhou 313001, China; 3 Department of Microtechnology and Nanoscience, Chalmers University of Technology, 41296 Goteborg, Sweden

**Keywords:** 2D/3D perovskites, photodiodes, defect passivation, dark current, high detectivity

## Abstract

Due to high responsivity and wide spectral sensitivity, metal halide perovskite photodiodes have a wide range of applications in the fields of visible light and near-infrared photodetection. Specific detectivity is an important quality factor for high-performance perovskite-based photodiodes, while one of the keys to achieving high detectivity is to reduce dark current. Here, 3-fluoro phenethylammonium iodide (3F-PEAI) was used to passivate the perovskite surface and form the two-dimensional (2D) perovskite on the three-dimensional (3D) perovskite surface. The as-fabricated passivated perovskite photodiodes with 2D/3D hybrid-dimensional perovskite heterojunctions showed two orders of magnitude smaller dark current, larger open circuit voltage and faster photoresponse, when compared to the control perovskite photodiodes. Meanwhile, it maintained almost identical photocurrent, achieving a high specific detectivity up to 2.4 × 10^12^ Jones and over the visible-near-infrared broadband photodetection. Notably, the champion photoresponsivity value of 0.45 A W^−1^ was achieved at 760 nm. It was verified that the 2D capping layers were able to suppress trap states and accelerate photocarrier collection. This work demonstrates strategic passivation of surface iodine vacancies, offering a promising pathway for developing ultrasensitive and low-power consumption photodetectors based on metal halide perovskites.

## 1. Introduction

In recent years, the organic-inorganic hybrid perovskites have brought a breakthrough in the optoelectronic applications, such as solar cells [1,2], light-emitting diodes [3,4], and photodetectors [5,6,7]. Their large optical absorption coefficient, long carrier diffusion length, high carrier mobility, and tunable bandgap enable photodetectors to show high photoresponsivity and fast response/recovery for various wavelength detection [8,9,10,11,12,13]. Notably, one of the key quality factors for a sensitive photodetector is specific detectivity (D*), which is proportional to the reciprocal of the noise-equivalent power (NEP) and is determined by the ratio of the responsivity over noise current [14]. Typically, the reverse bias dark current (I_dark_) contributes to the major noise current in realistic photodiodes [15,16,17,18]. However, to date, the solution-treated perovskite photodiodes have still suffered from relatively high I_dark_, lowering D* and thus restricting detection of weak light signals. Thereby, reducing I_dark_ is essential to facilitate their photodetection applications.

As reported, I_dark_ in photodiodes can mainly originate from the defects present within the perovskites and the minority charges injected from the electrodes under reverse bias [19,20]. Thus, the main strategies to suppress I_dark_ can be categorized into two groups: refinement of device configuration design and improvement of material crystal quality of light absorbing layers [21]. For example, hole/electron blocking layers and edge-covering layers have been introduced [20,22]. Fang et al. replaced poly(3,4-ethylenedioxythiophene):poly(styrenesulfonate)(PEDOT:PSS)withN4,N4′-bis(4-(6-((3-ethyloxetan-3-yl)methoxy)hexyl)phenyl)-N4,N4′-diphenylbiphenyl-4,4′-diamine (OTPD) to increase electron injection barrier from 1.1 eV to 2.5 eV and thus succeeded in suppressing I_dark_ [23]. Shen et al. created a large hole injection barrier (>2 eV) between the metal copper (Cu) electrode and the buckminsterfullerene (C_60_) layer by optimizing the contact interface [20]. Gelinck et al. developed inherent mechanisms about the effect of charge blocking layers to reduce I_dark_ and lowered I_dark_ in near-infrared perovskite photodiodes by suppressing carrier injection and interfacial carrier generation [24]. In addition, efforts have also been made to improve the crystal quality of the absorber layer through various strategies including doping [25,26], thin film processing engineering [27] and surface passivation [28]. Zhao et al. introduced thioacetamide (C_2_H_5_NS) to the CsPbBr_3_ film. The uncoordinated Pb^2+^ defects on the surface were passivated, suppressing nonradiative recombination and accelerating carriers extraction [29]. Jiang et al. used organic halide salt phenethylammonium iodide (PEAI) on perovskite films, and they inferred that the PEAI coating on the perovskite layer fills the iodine vacancies on the surface and at the grain boundary, resulting in passivation of the surface defects [30]. Inspired by the previous studies, the way guaranteeing both good crystal quality and optimized energy-band alignment can be very effective for sensitive perovskite photodiodes.

Two-dimensional (2D) metal halide compounds are a class of layered materials with atomic-level thicknesses, typically composed of metal cations and halogen anions (e.g., Cl^−^, Br^−^, I^−^). Due to their unique electronic, optical, and structural properties, these materials have attracted extensive attention in the fields of optoelectronics, energy and catalysis in recent years [31]. Among them, 2D perovskites are the most studied materials due to their resistance to oxygen and moisture compared to three-dimensional (3D) perovskites. In addition, two-dimensional/three-dimensional (2D/3D) hybrid-dimensional perovskite heterojunctions have also attracted strong interest, since the 2D layer plays a crucial role not only in passivating surface defects but also in facilitating carrier extraction [32,33,34,35]. Lai et al. ensured the reduction of surface iodine vacancy-induced trap states and favorable energy level alignment at the perovskite/hole transport layer interface by introducing a mixed-phase 2D perovskite to 3D perovskite [36]. Applying organic salts, which can form 2D perovskite through reaction with 3D perovskite, to a 3D surface, in principle, should allow us to decrease I_dark_ by reducing undesirable defects and improving energy-band alignment quality.

In this study, the 2D perovskite was introduced to the 3D perovskite/electron transport layer (ETL) interface using 3F-PEAI post-processing. To reduce electron blocking in the 2D perovskite between the 3D perovskite and the ETL, the 2D perovskite containing *n* ≥ 3 layers was selected, which possessed closer conduction band minimum (CBM) to the 3D perovskite. After 2D post-treatment, the scanning electron microscopy (SEM) and atomic force microscopy (AFM) images showed smoother surface morphology and the photoluminescence (PL) spectra showed the higher emission intensity. These results suggest that unwanted defects were effectively mitigated. By using surface defect passivation of the molecules, the I_dark_ of the photodiode was significantly reduced and a competitive D* of 2.4 × 10^12^ Jones was achieved. Evidently, this study demonstrated that defect passivation in perovskite thin films is one of the keys to achieving optimal performance of perovskite photodiodes.

## 2. Materials and Methods

### 2.1. Material Synthesis

Cesium iodide (CsI, >99.5%), lead iodide (PbI_2_, >99.5%), formamidinium iodide (FAI, >99.5%), methylammonium iodide (MAI, >99.5%), 3-fluoro phenethylammonium iodide (3F-PEAI, >99.5%), bathocuproine (BCP), Nickel oxide (NiOx, 2.5 wt.%), and poly(3,4-ethylenedioxythiophene):poly(styrenesulfonate) (PEDOT:PSS) were purchased from Xi’an Polymer Light Technology (Xi’an, China). Fullerene (C_60_) (99.5%), dimethyl sulfoxide (DMSO, anhydrous), dimethylformamide (DMF, anhydrous), isopropyl alcohol (IPA, anhydrous), and chlorobenzene (CB, anhydrous) were obtained from Aladdin (Tokyo, Japan). Indiumtin oxide (ITO) coated glasses (~15 Ω sq^−1^) were purchased from GuoKe JingYan (Tianjin) Technology (Tianjin, China). All of the chemicals and materials were used as purchased without further purification.

### 2.2. Device Fabrication

The ITO substrates were sequentially cleaned with detergent, deionized water, acetone, IPA, and ethanol by sonication treatment for 15 min in each step. After N_2_ drying, the ITO substrates were treated with O_2_ plasma for 15 min. For devices with EBL of PEDOT:PSS, the PEDOT:PSS layer was deposited by spin-coating at 4000 r.p.m. for 40 s and then annealed at 150 °C for 15 min. For devices with EBL of NiO_x_, the NiO_x_ layer was deposited by spin-coating at 5000 r.p.m. for 30 s and then annealed at 150 °C for 30 min. The 1.4 M FA_0.95_Cs_0.05_PbI_3_ perovskite precursor was prepared by dissolving 18.2 mg CsI, 645.4 mg PbI_2_, and 228.4 mg FAI in mixed solvents of DMF and DMSO (*v*/*v*, 4/1) stirring overnight. The precursor solution was spin-coated on the ITO/NiO_x_ substrates at 2000 r.p.m. for 10 s and then at 4000 r.p.m. for 40 s. During the second spin-coating step, 150 µL CB was dropped onto the perovskite film at 5 s before the end of the programme. The wet perovskite films were annealed at 100 °C for 30 min. For post-treated films, 2D ligand solution was prepared by dissolving 3F-PEAI (1 mg mL^−1^) and MAI (0.5 mg mL^−1^) in mixed solvents of IPA and DMF (*v*/*v*, 200/1). The 2D layer was fabricated by depositing the 2D ligand solution (150 µL) onto the perovskite film surface. After deposition, the film was spun at a rate of 4000 r.p.m. for 40 s with a 4000 r.p.m. per second acceleration. The film was then annealed at 100 °C for 5 min. Then ETL C_60_ (40 nm), BCP (8 nm) and Ag (70 nm) electrode were deposited by thermal evaporation in order.

### 2.3. Characterization and Measurement

The X-ray diffraction (XRD) patterns were performed using D2 PHASER (Bruker, Billerca, MA, USA). The steady-state PL and AFM measurements were performed using WITec Alpha300 Raman (WITec, Ulm, Germany). The morphology of the perovskite films was determined by SEM using Zeiss Sigma500 (Zeiss, Munich, Germany). The I−V curves of the photodiodes were obtainedusing Keithley 4200 (Keithley, Beaverton, OR, USA). Light sources with varied wavelengths were supplied using NKT Photonics SuperK SELECT (NKT Photonics, Copenhagen, Denmark). The response speed of the photodiodes was evaluated by recording the transient signal to light pulses with variable frequenciesusing FS-PRO (Primaries, Shanghai, China). The light pulses were generated by a laser diode of 650 nm that was connected to a signal generator.

## 3. Results and Discussion

Considering the organic-inorganic hybrid perovskite FA_0.95_Cs_0.05_PbI_3_ has good thermal stability and suitable bandgap, it was selected as the absorber layer of a near-infrared photodetector in this work [37]. The pristine FA_0.95_Cs_0.05_PbI_3_ perovskite films were prepared by a one-step spin-coating method. Due to dipole moment and hydrophobic nature, the fluorinated ammonium salts are effective additives to improve device performance. In addition, they are able to produce 2D/3D heterojunctions containing more *n* = 3 2D perovskite than those formed by PEAI [38]. Since the fluorinated ammonium salts are effective additives to improve the device performance possibly due to their dipole moment and hydrophobic nature, 2D 3F-PEAI was chosen to spin-coat on the top of the 3D perovskite film for cation exchange. It enabled the passivation of defects on the 3D perovskite surface [39,40,41,42]. Notably, introducing a small amount of DMF and MAI to precursor solution of 3F-PEAI was able to improve device performance due to better energy-band alignment [38]. Thus, this precursor solution was spin-coated onto the 3D perovskite film to prepare 2D perovskite layers, forming 2D/3D hybrid-dimensional perovskite heterojunctions.

**Figure 1:**
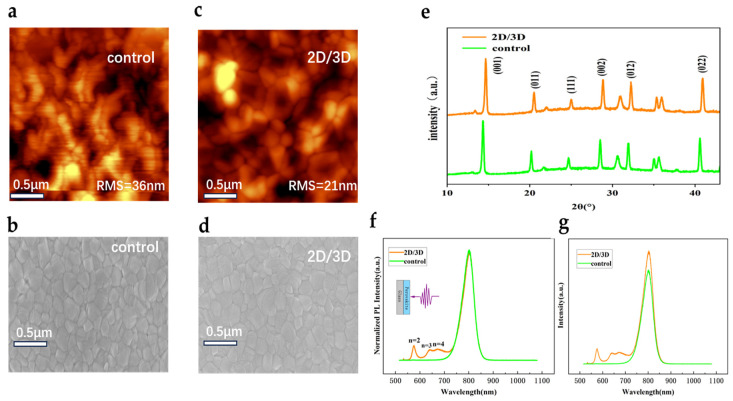
(**a**,**b**) AFM and SEM images of the control perovskite film; (**c**,**d**) AFM and SEM images of the passivated perovskite film; (**e**) the XRD patterns of the control and the passivated perovskite films. (**f**) Normalized steady-state PL spectra of the control and the passivated perovskite films, showing 2D features after passivation; (**g**) PL spectra of the control and passivated perovskite thin films. Subsequently, the perovskite photodiodes were fabricated with the inverted structure (that is, positive-intrinsic-negative, or PIN) because of the generally better stability [44,45,46,47]. The perovskite photodiodes were fabricated on the glass substrates with patterned ITO electrodes on top, as shown in Figure 2a. The ITO electrodes were first coated with an electron-blocking layer (EBL) of NiOx, and then FA_0.95_Cs_0.05_PbI_3_ perovskite was prepared to form absorber layer, followed by the sequential deposition of fullerene (C_60_)/BCP as ETL, and finally the Ag electrode was deposited. The crossover ITO and Ag electrodes determined the active region area to be 0.02 cm^2^.

To examine the microstructure passivation effects of the as-synthesized perovskite films, SEM and AFM characterization were carried out. As shown in Figure 1a,b, the control film without 2D layer exhibited many distinct grain boundaries, possessing a large root-mean-square (RMS) roughness of 36 nm. In comparison, the post-treated film, containing the 2D/3D hybrid-dimensional heterojunctions, exhibited fewer grain boundaries and the smaller RMS roughness value down to 21 nm in Figure 1c,d, and the surface morphology became smoother. Evidently, it can be inferred that introduction of 2D perovskites was able to passivate surface and suppress grain boundaries, which can potentially reduce the recombination loss. Figure 1e illustrates the characteristic X-Ray diffraction (XRD) patterns of the control and the passivated perovskite films, while the 2D phase cannot be observed directly in the XRD result. This was consistent with the previously reported results [19,36].

Furthermore, to verify the existence of 2D perovskite on the surface, steady-state PL measurements were performed. The normalized PL spectra of the control and the passivated perovskite films are shown in Figure 1f. In addition to the PL peaks from 3D perovskites at around 800 nm, PL peaks associated with *n* = 2, 3, and 4 were also observed in the passivated films, indicating the presence of the 2D perovskites on the surface [19,43]. Meanwhile, the stronger PL peak intensity of the passivated perovskite films in Figure 1g indicated the trap-assisted carrier recombination on the surface can be effectively suppressed, further illustrating the reduced surface defects.

**Figure 2:**
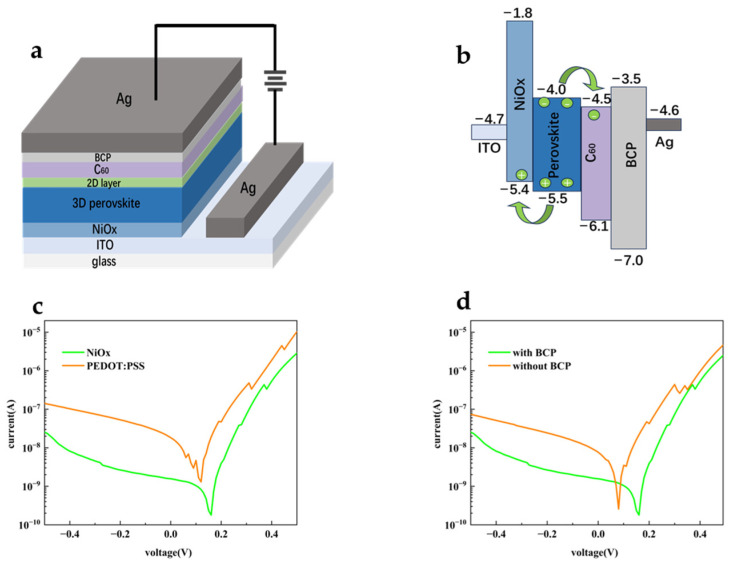
(**a**) Schematic device structure of the perovskite photodiode, fabricated with the inverted structure; (**b**) The energy level diagram of the perovskite photodiode; (**c**) I_dark_ vs. applied voltages for perovskite photodiodes using different EBLs of PEDOT:PSS and NiOx, respectively; (**d**) I_dark_ vs. applied voltages for perovskite photodiodes with and without BCP.

Figure 2b displays the energy level diagram of the perovskite photodiode. NiOx/FA_0.95_Cs_0.05_PbI_3_/C_60_ type II heterojunctions were formed, which can potentially mitigate the interfacial complexation and promote the separation of photogenerated carriers. As displayed in Figure 2c, the I_dark_ of the devices was greatly reduced compared to the devices using PEDOT:PSS. Compared with the common EBL of PEDOT:PSS, the higher electron injection barrier at the ITO/NiO_x_ interface can significantly block the minority electron injection from ITO under reverse bias. In addition, the deep highest occupied molecular orbital (HOMO) of BCP achieved hole blocking from the silver electrode. As expected, Figure 2d shows the current-voltage (I-V) characteristics of the devices in the dark before and after the incorporation of BCP. An order of magnitude reduction in I_dark_ was achieved with BCP, which was indicative of the hole-blocking and electron-transport functions of the BCP layer. Therefore, NiOx and BCP were employed in the photodiodes.

**Figure 3:**
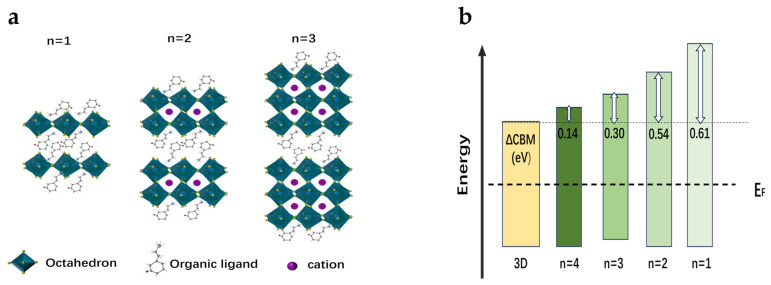
(**a**) Schematic of *n* = 1, 2 and 3 2D structures based on lead iodine perovskite. (**b**) Band alignment of 3D and 2D perovskites films with different *n* values.

In terms of previously reported work, many well-performing photodiodes used a 2D/3D heterostructure, fabricated via spin coating organic salts onto the surface of 3D perovskites [36]. It was found that this heterostructure boosted performance via enhanced electron blocking. It was beneficial for the negative-intrinsic-positive (NIP) device architecture, in which the 2D-treated perovskite surface was coated with an EBL. However, in this work, such strategy can hinder carrier transport, because 2D-treated perovskite surface was coated with an ETL. It is worth noting that 2D perovskites are perovskite quantum well layers confined between large organic ligands [48]. The number of stacked lead (Pb) octahedra between the organic spacers determines the width of the 2D layer: *n* = 1, 2, 3 and so on, as exhibited in Figure 3a. As reported, the CBM of 2D perovskite moves down as the increase of n while the valence band maximum (VBM) is almost unchanged (Figure 3b) [38]. To passivate surface defects and reduce resistance to carrier transport, it is rational to deduce that a capping layer of *n* ≥ 3 2D perovskites would be beneficial for PIN photodiode performance due to their deeper CBM. As reported, the width of the 2D layer can be modulated by changing the ratio of the 2D ligands to the 3D perovskite precursors in solution [49]. Thereupon, MAI and DMF were introduced to precursor solutions to produce 2D perovskite capping layers containing *n* ≥ 3 layers. MAI was used to reduce the ratio of 2D ligands to A-site cation and DMF introduced Pb of 3D perovskite surface to 2D perovskite layer [38]. This precursor solution was spin-coated onto the 3D perovskite film, forming the 2D/3D hybrid-dimensional perovskite heterojunctions. In this way, it was thus expected that the passivated perovskite photodiodes can achieve a good trade-off between defect passivation and carrier transport.

**Figure 4:**
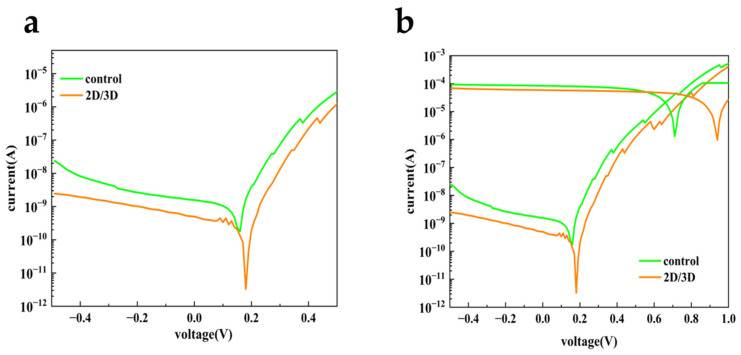
(**a**) I_dark_ versus applied voltages for the control perovskite photodiode and the passivated perovskite photodiode; (**b**) I–V characteristics in the dark and under illumination of 800 nm light for the control and the passivated perovskite photodiodes, respectively.

To systematically investigate the impact of 2D/3D hybrid-dimensional perovskite heterojunctions on photodetection capability, the photodiode incorporating this structure and the control device were studied below, respectively. Figure 4a shows the I–V characteristics of perovskite photodiodes in the dark for voltages ranging from −0.5 V to 0.5 V before and after passivation, respectively. It is worth noting that photodiodes after passivation (with 2D/3D heterojunctions) showed much smaller I_dark_ under reverse bias. As illustrated, the minimum value down to 3.2 × 10^−12^ A of the I_dark_ for the photodiodes with 2D/3D heterojunctions was obtained at 0.18 V, which was two orders of magnitude smaller than that of the control one. The reduced I_dark_ allows the passivated device to detect very weak light, contributing to photodetection sensitivity and signal to noise ratio (SNR). It was reported that the surface defects caused by iodine vacancies mainly contribute to the I_dark_ in the perovskite photodiodes [19]. Together with the former AFM and PL results shown in Figure 1, it can be reasonably deduced that the 2D capping layers successfully passivated the surface defects and suppressed the generation-recombination current density in the dark according to the Shockley–Read–Hall mechanisms. In terms of the non-zero I_dark_ at 0 V bias, this might be associated with different rates of carrier injection from the electrodes, leading to accumulation of excess carriers. Meanwhile, the capacitance increased with accumulation of excess carriers, which could result in the non-zero I_dark_ at 0 V bias [50].

In addition, to inspect the near-infrared detection performance of the photodiodes, photocurrent curves of the perovskite photodiodes under illumination of 800 nm light are shown in Figure 4b. It can be seen that both devices show excellent photoresponse, while the passivated one maintained the almost identical photocurrent with smaller I_dark_ and larger open circuit voltage (V_OC_). Namely, the device with 2D/3D heterojunctions was able to deliver more sensitive near-infrared photodetection, lower power consumption and more prominent self-powered work mode. Moreover, the photoresponse behavior also indicated that the resistance to carrier transport of 2D perovskites was reduced due to *n* ≥ 3 of the 2D capping layer. Usually, the quantum confinement effect would upshift the CBM of the 2D perovskites, resulting in the electron blocking between the 3D perovskite and the EHL. Nevertheless, in this work the electron blocking can be ignored because of the introduction of *n* ≥ 3 2D perovskites, whose CBM was very close to that of the 3D perovskites.

**Figure 5:**
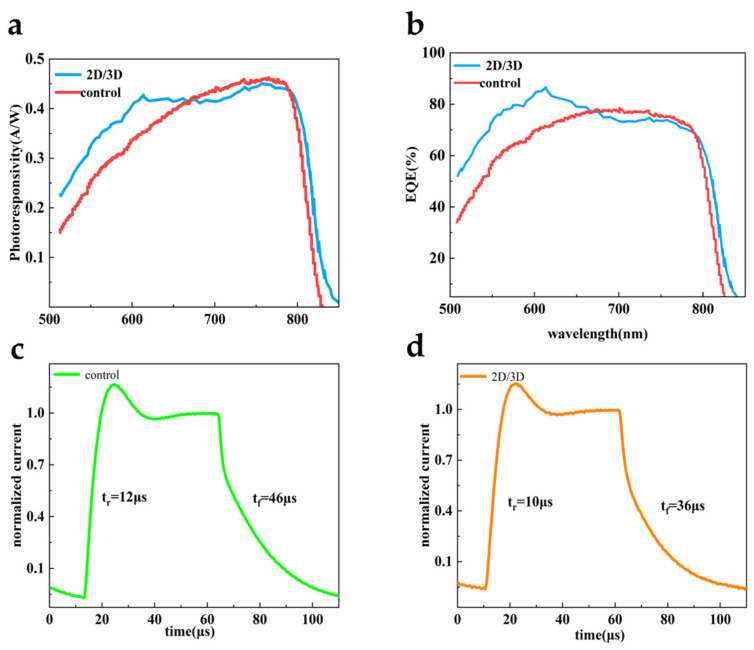
(**a**) Photoresponsivity spectra of the control and the passivated photodiodes; (**b**) The EQE spectra of the control and the passivated photodiodes; (**c**,**d**) normalized transient photocurrent response at 0 V for the control and the passivated perovskite photodiodes, respectively.

To further demonstrate photodetection performance, Figure 5a shows the wavelength-dependent responsivity of the control and passivated devices working with the self-powered mode, measured at zero bias. The passivated device showed high photoresponsivity over the visible-near-infrared broadband region (500 nm–800 nm), and its cut-off wavelength was ~820 nm. As can be seen, the photoresponsivity of the passivated device was enhanced, but it decreased slightly in the wavelength range of 670 nm~790 nm, which may be due to the electron blocking of the *n* = 2 2D perovskite [38]. Although this partial decrease of photoresponsivity was existed in the specific range, the D* value of the passivated device would still be larger than that of the control one, because of the obviously restrained I_dark_. Notably, the champion photoresponsivity value of 0.45 A W^−1^ was achieved at 760 nm for passivated device. The external quantum efficiency (EQE) is another important parameter for photodetectors, which can be estimated by the following equation:

(1) $$EQE=\frac{Rhc}{e\lambda },$$

where *h* is the Planck’s constant, *c* is the light speed, and *e* is the electron charge [6]. As illustrated in Figure 5b, the 2D/3D device delivers a maximum EQE up to 86.6% at zero bias, while the maximum EQE of the control one was 78.4%. Assuming that shot noise from the I*_dark_* is the major contributor to the total noise, the D* can be estimated by the following equation:

(2) $$D^{*}=R\times \sqrt{\frac{A}{2eI_{dark}}}$$

where *A* is the active area of the device and *R* is the photoresponsivity. When under 760 nm illumination, the passivated photodiodes achieved the highest D* of 2.4 × 10^12^ Jones due to the low I*_dark_*. Additionally, photoresponse speed of the devices under 0 V is evaluated in Figure 5c and 5d, respectively. As illustrated, the passivated photodiode exhibited faster response with rise time of 10 μs and fall time of 36 μs, compared to rise time of 12 μs and fall time of 46 μs for the control photodiode. This difference can be attributed to weak electron blocking between 3D perovskite and 2D capping layers. In addition, the defects on the surface of 3D perovskite can also cause trapping and detrapping processes, resulting in slow response speed in photodiodes. Thus, reduced response time further indicated that the 2D layers effectively passivated the defects and accelerated the photocarrier collection process.

## 4. Conclusions

In summary, the passivated perovskite photodiodes with 2D/3D hybrid-dimensional heterojunctions were fabricated, showing high-detectivity, broadband photodetection and the self-powered mode. Significantly, using the 3F-PEAI surface treatment, the 2D perovskite thin layer was formed on the surface of its 3D bulk. In addition, the 2D perovskite capping layers contained *n* ≥ 3 layers, possessing closer CBM to 3D perovskites and weak electron blocking between 3D perovskites and the ETL. Notably, the surface iodine-vacancy-induced defects were effectively passivated by the 2D perovskites. Consequently, the perovskite photodiodes after passivation exhibited a minimum I_dark_ down to 3.2 × 10^−12^ A at 0.18 V, a high peak photoresponsivity of 0.45 A W^−1^ at zero bias and 760 nm light illumination, a high peak D* up to 2.4 × 10^12^ Jones and rapid rise/fall time of 10/36 µs. Moreover, the passivated photodiodes were expected to have better environmental stability because of the moisture resistance of 2D perovskites. This study establishes a defect engineering approach through targeted passivation of surface iodine vacancies in metal halide perovskites, which effectively suppresses trap states, optimizes surface/interface charge transport and helps to improve perovskite photodetection sensitivity.

## Data Availability

The data that support the findings of this study are available from the corresponding author upon reasonable request.
